# Use of Mesothelial Cells and Biological Matrices for Tissue Engineering of Simple Epithelium Surrogates

**DOI:** 10.3389/fbioe.2015.00117

**Published:** 2015-08-17

**Authors:** Christian Claude Lachaud, Berta Rodriguez-Campins, Abdelkrim Hmadcha, Bernat Soria

**Affiliations:** ^1^Andalusian Center for Molecular Biology and Regenerative Medicine – Centro Andaluz de Biología Molecular y Medicina Regenerativa (CABIMER), Seville, Spain; ^2^Centro de Investigación en Red sobre Diabetes y Enfermedades Metabólicas (CIBERDEM), Madrid, Spain; ^3^Departamento de I+D, New Biotechnic S.A., Seville, Spain; ^4^Fundación Andaluza de Investigación y Desarrollo (FAID), Seville, Spain

**Keywords:** tissue engineering, epithelial surrogates, biological matrices, biomaterials, simple epithelia, mesothelial cells, serosal membranes, corneal endothelium

## Abstract

Tissue-engineering technologies have progressed rapidly through last decades resulting in the manufacture of quite complex bioartificial tissues with potential use for human organ and tissue regeneration. The manufacture of avascular monolayered tissues such as simple squamous epithelia was initiated a few decades ago and is attracting increasing interest. Their relative morphostructural simplicity makes of their biomimetization a goal, which is currently accessible. The mesothelium is a simple squamous epithelium in nature and is the monolayered tissue lining the walls of large celomic cavities (peritoneal, pericardial, and pleural) and internal organs housed inside. Interestingly, mesothelial cells can be harvested in clinically relevant numbers from several anatomical sources and not less important, they also display high transdifferentiation capacities and are low immunogenic characteristics, which endow these cells with therapeutic interest. Their combination with a suitable scaffold (biocompatible, degradable, and non-immunogenic) may allow the manufacture of tailored serosal membranes biomimetics with potential spanning a wide range of therapeutic applications, principally for the regeneration of simple squamous-like epithelia such as the visceral and parietal mesothelium vascular endothelium and corneal endothelium among others. Herein, we review recent research progresses in mesothelial cells biology and their clinical sources. We make a particular emphasis on reviewing the different types of biological scaffolds suitable for the manufacture of serosal mesothelial membranes biomimetics. Finally, we also review progresses made in mesothelial cells-based therapeutic applications and propose some possible future directions.

## Introduction

Tissue engineering has emerged as a promising alternative to conventional medicine to achieve the healing and regeneration of human damaged tissues and organs. The manufacture of functionally optimal bioartificial tissues is an extremely complex process relying on a comprehensive stepwise combination of cells with scaffolds, extracellular matrices (ECM), and molecular signals. The achievement of such objective usually relies on the combination of advanced knowledge and skills from interdisciplinary specialists, making tissue engineering one of the most challenging fields of biomedical research.

Elaborated bioartificial soft tissues and inclusively some bioartificial organs are under current experimental development and evaluation in laboratories (Mikos et al., 2006; Atala, 2009; Atala et al., 2012). Unfortunately, their clinical application in humans is still far away from being accessible and to date only a limited number of bioartificial tissues including skin substitutes (Debels et al., 2015) or bioprosthetic aortic valves (Brown et al., 2009) have been successfully transferred to clinical practice in humans.

The majority of tissue-engineering applications aiming at regenerating complex human tissues remain still a great challenge, principally because they should be functionally prevascularized to be efficiently integrated into the host (Nomi et al., 2002; Novosel et al., 2011). This has been principally the case for large 3D bioartificial tissues performed so far and that acquired only superficial neovascularization by the surrounding host tissues vasculature during their initial stage of implantation in animal models (Nomi et al., 2002; Park and Gerecht, 2014). To solve this issue, a host of experimental therapeutic angiogenic strategies involving endothelial progenitor cells, proangiogenic growth factors, bioactive microcarriers, or preformed bioartificial vascular networks among others are currently under intense research to achieve the generation of functional prevascularized neotissues [for specific review, see Kaully et al. (2009), Lovett et al. (2009), Roy et al. (2011), and Park and Gerecht (2014)].

In sharp contrast with complex 3D vascularized tissues are avascular monolayered tissues such as simple epithelia, which are made up of a single layer of simple epithelial cells anchored onto a thin basement membrane. Simple epithelia are basically subclassified as columnar, cuboidal, or squamous depending on the size and shape of their cellular component. Simple squamous epithelia are highly distributed within the adult body. Its major form, the mesothelium is the monolayered tissue lining the walls of the largest celomic cavities (pleural, pericardial, and peritoneal) and the surface of celomic visceral organs (Mutsaers, 2002). A layer of mesothelial cells is additionally found lining the side of the Reissner’s membrane facing the scala vestibuli within the cochlea. Interestingly, other tissues such as the vascular endothelium, corneal endothelium, and also the synovial membrane lining display in diverse degrees morphological hallmarks as well as structural and biochemical markers consistent with simple epithelial cells (reviewed in Table 1).

**Table 1:** **Phenotypic marker profiles of different types of simple epithelial-like cells**.

Markers	Vascular endothelial cells	Corneal endothelial cells	Fibroblast-like synoviocytes	Mesothelial cells
Vimentin	++ (Chung-Welch et al., 1997b)	++ (Vazquez et al., 2015)	++ (Bartok and Firestein, 2010)	++ (Lachaud et al., 2014a)
N-cadherin	++ (Chung-Welch et al., 1997b)	++ (Zhu et al., 2008b; Lachaud et al., 2014b)	++ (Agarwal et al., 2008)	++ (Lachaud et al., 2014b)
Pan-Cytokeratin	− (Chung-Welch et al., 1997b)	?	?	++ (Yanez-Mo et al., 2003)
Cytokeratin 18	− (Chung-Welch et al., 1997b)	++ (Merjava et al., 2009)	?	++ (Chung-Welch et al., 1997b)
Mesothelin	?	++ (Lachaud et al., 2014b)	?	+ (Lachaud et al., 2014b)
WT1	−/+* (Wagner et al., 2008; Duim et al., 2015)	?	?	++ (Lachaud et al., 2014a)
E-cadherin	−	+ (Zhu et al., 2008b)	− (Agarwal et al., 2008)	−/+* (Lachaud et al., 2014a)
VE-cadherin	++ (Tsai et al., 2007; Chlupac et al., 2014)	+*/+ (Zhu et al., 2008b; Huang et al., 2010b)	?	−
ZO-1	++ (Medina et al., 2010; Shao et al., 2011)	++ (Zhu et al., 2008b; Lachaud et al., 2014b)	++	++ (Lachaud et al., 2013, 2014a)
β-catenin	++ (Medina et al., 2010)	++ (Zhu et al., 2008b; Lachaud et al., 2014b)	++ (Xiao et al., 2011)	++ (Lachaud et al., 2013, 2014a)
Aquaporin 1	++ (Mobasheri and Marples, 2004)	++ (Chng et al., 2013)	+ (Mobasheri and Marples, 2004)	+ (Lai et al., 2001; Ji and Nie, 2008)
COL8A1	+ (Muragaki et al., 1991)	++ (Chng et al., 2013)	?	?
COL8A2	+ (Muragaki et al., 1991)	++ (Chng et al., 2013; Lachaud et al., 2014b)	?	++ (Lachaud et al., 2014b)
SLC4A4	− (Romero et al., 2013)	++ (Chng et al., 2013; Lachaud et al., 2014b)	?	++ (Lachaud et al., 2014b)
SLC4A11	?	++ (Damkier et al., 2007)	?	?
CA-II	++ (Su et al., 2004)	++ (Chng et al., 2013; Lachaud et al., 2014b)	?	++ (Lachaud et al., 2014b)
NA^+^/K^+^-ATPase	++ (Trevisi et al., 2006)	++ (Lachaud et al., 2014b; Vazquez et al., 2015)	?	++ (Witowski et al., 1997; Lachaud et al., 2014b)
CD166 (ALCAM)	++ (Swart, 2002)	++ (Okumura et al., 2014)	++ (Joo et al., 2000)	++ (Ross et al., 1998)
vWF (Factor VIII)	++ (Tsai et al., 2007; Chlupac et al., 2014)	−/+ (Shamsuddin et al., 1986)	− (Schwachula et al., 1994)	+* (Chung-Welch et al., 1997b)
CD31 (PECAM-1)	++ (Tsai et al., 2007; Chlupac et al., 2014)	− (Shamsuddin et al., 1986; Huang et al., 2010b)	− (Schwachula et al., 1994)	− (Lachaud et al., 2013, 2014a)
Dil-Ac-LDL	++ (Chung-Welch et al., 1997b; Medina et al., 2010)	− (Huang et al., 2010b)	+ (Schwachula et al., 1994)	+* (Chung-Welch et al., 1997b)
CD45 (LCA)	− (Medina et al., 2010)	− (Huang et al., 2010b)	− (Tran et al., 2008)	− (Lachaud et al., 2013, 2014a)
CD29 (integrin β1)	++ (Chlupac et al., 2014; Kawasaki et al., 2015)	?	?	++ (Lachaud et al., 2013, 2014a)
CD106 (VCAM-1)	++ (Su et al., 2004)	− (Foets et al., 1992)	+*/+ (Bombara et al., 1993; Bartok and Firestein, 2010)	− (Lachaud et al., 2013, 2014a)
CD44 (HCAM)	+ (Su et al., 2004)	− (Foets et al., 1992)	++ (Schwarting et al., 1996)	+ (Lachaud et al., 2013, 2014a)
CD90 (Thy-1)	++ (Su et al., 2004)	?	++ (Bartok and Firestein, 2010)	+/++ (Lachaud et al., 2014a)
CD54 (ICAM-1)	++ (Murohara et al., 1999)	+ (Foets et al., 1992)	++ (Bartok and Firestein, 2010)	++ (Lachaud et al., 2013, 2014a)

Markers expression levels: −, negative; +*, very weak; +, intermediate; ++, strong. /, this symbol is used when expression varies between distinct published works. For example, −/+* indicates a variation in reported expression ranging from negative to weak. ?, this symbol is used for unknown expression levels.

*ALCAM, activated leukocyte cell adhesion molecule; CA-II, carbonic anhydrase II; COL8A1, collagen type VIII, alpha 1; COL8A2, collagen type VIII, alpha 2; HCAM, homing cell adhesion molecule; ICAM-1, intercellular cell adhesion molecule 1; NA+/K+-ATPase, sodium–potassium adenosine triphosphatase; PECAM-1, platelet endothelial cell adhesion molecule 1; SLC4A4, electrogenic sodium bicarbonate cotransporter 1; SLC4A11, sodium bicarbonate transporter-like protein 11; VCAM-1, vascular cell adhesion molecule 1; vWF, von Willebrand factor; WT1, Wilms tumor protein; ZO-1, zona occludens 1*.

There has been a growing consensus about the concept that these distinct tissues could somehow be considered other forms of simple squamous epithelia. The synovial membrane lining, mesothelium, vascular endothelium, and corneal endothelium are each of them endowed with specific functions and distinct endogenous regenerative capacities, which is high for the mesothelium (Mutsaers et al., 2007), intermediate for the vascular endothelium (Toya and Malik, 2012) and barely inexistent for the corneal endothelium (Bourne, 2003). Despite such divergences, the inherent regenerative capacity of some forms of simple epithelia is however strongly linked to the extent of their damage. In some instances, accidents, surgical trauma, or diseases can lead to their irreparable damage and ultimately to their loss of function. Their reconstruction or substitution can be eventually achieved through transplantation of autologous or allogeneic native tissues, the accessibility of which is however limited by the important shortage of suitable donor tissues. Tissue engineering of artificial tissues biomimetics has emerged as a promising alternative to the lack of native replacement tissues. The use of mesothelial cells in tissue engineering of simple epithelial-like tissues such as the vascular endothelium or the corneal endothelium has been already initiated and is technically accessible. Sources of mesothelial cells and appropriate biological scaffolds for these applications have been already identified. Additionally, the transdifferentiative capacity of mesothelial cells is also better understood and controlled and should be taken in account in the tissue-engineering procedures of these bioartificial tissues.

Herein, we provide an overview of the main biological and biochemical properties of mesothelial cells and detail their different clinical sources. A particular emphasis is done on reviewing biological scaffolds suitable for tissue engineering of simple epithelial-like tissues. Finally, we review the use of mesothelial cells in different tissue-engineering applications and also suggest some possible future directions.

## Mesothelium: Structure and Functions

The single layer of flattened cells covering celomic serous membranes was first described by Bichat in 1827 and later on termed “mesothelium” by Minot in 1880 in reference to its mesodermal origin. Although the mesothelium cells layer appears to be morphostructurally similar between different anatomical locations at a macroscopic level, posterior studies however led to the evidence that parietal mesothelial cells show morphological and biochemical differences with visceral mesothelial cells when examined at an ultrastructural level (Michailova et al., 1999). Parietal mesothelial cells which are the cells lining the walls of celomic cavities are rather large and flattened polygonal cells with low-to-intermediate intracellular organelles, whereas visceral mesothelial cells lining celomic organs are by contrast more tightly compacted and cuboidal and rich in intracellular organelles, principally mitochondria and rough endoplasmic reticulum (RER), indicative of their higher metabolic state (Mutsaers and Wilkosz, 2007).

Biostructurally, the mesothelium represents a semi-permeable laminar interface that separate fluid-filled body cavities from blood vessels and lymphatics running within the underneath submesothelial connective tissue layers. Besides its property of physical barrier, the mesothelium acts also as bioactive interface regulating fluid flows interchanges across its surface to maintain an optimal osmolarity and ionic activity of body cavity fluids; these functions are mainly accomplished by transmembrane ion pumps (Na^+^/K^+^-ATPase) and water channels (aquaporins) (Witowski et al., 1997; Ji and Nie, 2008). Among other main functions, mesothelial cells also secrete lubricants that stay electrochemically entrapped between their numerous surface microvilli to create a lubricated thin film or glycocalyx allowing the free sliding of opposite parietal and visceral mesotheliums with minimal abrasion (Mutsaers, 2002). The mesothelial glycocalyx is mainly composed by glycosaminoglycans, especially hyaluronan, a large anionic polymer of disaccharides with high hydrophilicity which forms a highly hydrated gel layer (Yung and Chan, 2007). Mesothelial cells also actively regulate celomic cavities homeostasis and inflammatory status via their secretion of numerous pro- and anti-inflammatory cytokines (Lanfrancone et al., 1992; Mutsaers and Wilkosz, 2007; Ji and Nie, 2008). It has also been evidenced that mesothelial cells are actively recruited during serosal regeneration, through processes of proliferation, migration or delamination, and secretion of a large variety of growth factors or cytokines (Foley-Comer et al., 2002; Mutsaers, 2002; Herrick and Mutsaers, 2004; Mutsaers, 2004; Mutsaers and Wilkosz, 2007; Carmona et al., 2011).

## Plasticity of Mesothelial Cells

Mesothelial cells are intriguing cells, because they are mesodermal in origin but however display phenotypic properties rather consistent with simple epithelial cells, an ambiguous phenotype that is reflected by their coexpression of both mesenchymal and epithelial lineage markers (Mutsaers, 2002; Herrick and Mutsaers, 2004; Mutsaers and Wilkosz, 2007).

The concept that the mesothelium may represent a primitive mesoderm was first postulated by Donna and Betta, based on their observation of areas of cartilaginous and osseous differentiation in malignant mesotheliomas (Donna and Betta, 1981; Debels et al., 2015), a tumor arising from mesothelial cells [for review, see Carbone et al. (2012)]. This concept was further reinforced by several lineage tracing studies conducted in the mouse embryo where mesothelial cells were found to originate stromal VSMCs and fibroblasts through a process of epithelial-to-mesenchymal transition in the developing heart, lung, gut, and liver (Dettman et al., 1998; Wilm et al., 2005; Cai et al., 2008; Que et al., 2008; Zhou et al., 2008; Asahina et al., 2011). Furthermore, embryonic mesothelial cells were also shown to generate stellate cells and a subset of cardiomyocytes in the developing liver and heart, respectively (Cai et al., 2008; Zhou et al., 2008; Asahina et al., 2011). Additionally, several *in vitro* differentiation studies demonstrated that adult mesothelial cells isolated from human and adult rodents could recapitulate an epithelial-to-mesenchymal transition and differentiate along the VSMCs, fibroblasts, chondrocytes, osteocytes, and adipocytes lineages when cultured upon adequate inductive conditions (van Tuyn et al., 2007; Lansley et al., 2011; Lachaud et al., 2013; Lachaud et al., 2014a). Consistent with these *in vitro* findings, a recent *in vivo* mesothelial lineage tracing study, conducted in the postnatal mouse, demonstrated that mesothelial cells covering the visceral adipose tissue are the precursor cells giving rise to white adipocytes (Chau et al., 2014). Furthermore, the ability of adult mesothelial cells to adopt myofibroblasts or inclusively macrophage-like features in response to pathological conditions of the peritoneal cavity may represent another evidence of their inherent plasticity and ability to switch their phenotype upon the microenvironment milieu (Yanez-Mo et al., 2003; Katz et al., 2011). Altogether, these studies provide converging evidence supporting the concept that adult mesothelial cells retain embryonic mesodermal multilineage differentiation capacity and could represent a population of primitive mesodermal stem cells. Their inherent plasticity is strongly supporting their use as cellular surrogate for tissue engineering of different types of specialized simple squamous epithelia.

## Immunomodulatory and Anti-Inflammatory Properties of Mesothelial Cells

The capacity of a cellular phenotype to reverse or ameliorate the clinical course of inflammatory diseases is of critical therapeutic relevance. Such capacity has been first described in mesenchymal stromal cells (MSCs) used in experimental animal models for human inflammatory diseases. Their protective effects was found to be largely attributed to their hypoimmunogenicity and capacity to regulate innate immune cells functions through secretion of soluble and membrane-bound factors with potent immunosuppressive and/or immunomodulatory activities [for review, see Glenn and Whartenby (2014)].

This major discovery has prompted a general interest in elucidating whether other cell types are endowed with similar properties. The first evidence that cells of the mesothelial lineage could display anti-inflammatory and immunosuppressive properties arose from studies of human malignant mesotheliomas, where it was found that mesothelial tumorigenic cells escape from the control of the immune system through suppression of the proliferation and functions of T lymphocytes and increased recruitment of immunosuppressive regulatory T cells (Hegmans et al., 2006). Later on, normal human omental mesothelial cells were found capable to potently suppress the proliferation of pro-inflammatory γδ T cells as well as of CD4^+^ and CD8^+^ T lymphocytes (T cells), through their secretion of the immunosuppressor TGF-β (Lin et al., 2013). A recent work also indicated that CD90^+^/CD45^−^ human mesothelial cells belonging to peritoneal fluid could immunosuppress CD4^+^ T cells *in vitro* through their potent expression of arginase I and consequent depletion of L-arginine, a major molecule required for T cells activation (Kitayama et al., 2014). Taking in account these *in vitro* results, it may therefore be expected that bioengineered artificial tissues performed with heterologous mesothelial cells should be globally hypoimmunogenic with a prognostic of good host-tissue integration.

## Clinical Sources of Mesothelial Cells

A critical issue in autologous cellular therapies is the identification of accessible anatomical sources from which can be harvested cells in therapeutically relevant numbers and with minimal health impact. In this way, the presence of several celomic cavities in the adult human body offers a large range of approaches (anatomical sources and procedures) to harvest mesothelial cells. Due to its largest size, the abdominal cavity is the predominant anatomical source from where mesothelial cells are harvested. Specific peritoneal sources and isolation procedures are reviewed below.

### Greater omentum

The greater omentum is broadly considered as an optimal and reliable source of mesothelial cells, principally because large pieces of this tissue can be surgically harvested with minimal health concerns and can provide clinically relevant numbers of mesothelial cells (Riera et al., 2006). In humans, the greater omentum or epiploon is the largest fold of peritoneum filled with abundant visceral adipose tissue with a surface area ranging from 300 to 1500 cm^2^. Like other visceral mesotheliums, the omental mesothelium displays a high cellularity and can provide around one million mesothelial cells from each square centimeter of omental tissue (Pronk et al., 1993). Furthermore, laparoscopy represents an effective minimal invasive approach to collect reduced pieces of omental tissue.

### Mesenteric membrane

The peritoneal cavity harbors several portions of serous membranes connecting parietal and visceral components. The mesentery is found to fully meet with such characteristics, and the mesentery is a reticular laminar structure resulting from a fold of the dorsal parietal mesothelium that enwraps in its extremity the overall length of the intestinal tract (Coffey, 2013). The mesenteric membrane acts as a connective supportive structure to hold the reticular network of intestinal vasculature, lymphatics, and nerves. Interestingly, some portions of the mesenteric membrane lack vasculature and associated perivascular adipose tissue, thus appearing as a transparent sheet composed of a double layer of mesothelium enclosing loosely arranged collagenous and elastic fibers (Coffey, 2013). Although previous studies already reported the isolation of rat and human mesenteric mesothelial cells for experimental research procedures (Chailley-Heu et al., 1997; Takazawa et al., 2005), their application in tissue engineering has not been reported. Despite this, it is reasonable to believe that laparoscopic surgery could allow the excision of small areas of avascular mesenteric membrane with minimal clinical impact and from which a relevant number of autologous or heterologous mesothelial cells could be isolated for tissue engineering of serosal membranes biomimetics.

### Peritoneal fluid

The peritoneal serosal fluid harbors a quite relevant population of free-floating cells, which were initially described as corresponding to resident macrophages and in lower extent to polymorphonuclear leukocytes and lymphocytes. Later studies however indicated that a significant subset of free-floating mesothelial cells normally coexists within the peritoneal fluid of healthy humans and rodents (Bercovici and Gallily, 1978; Stauffer et al., 1978). Interestingly, the prevalence of free-floating mesothelial cells strongly increases in the peritoneal fluid of patients undergoing continuous ambulatory peritoneal dialysis (CAPD) (Fok et al., 1989) and during serosal regeneration processes (Foley-Comer et al., 2002; Mutsaers et al., 2007).

Cultures of human peritoneal mesothelial cells could be successfully established from peritoneal lavage cells collected through laparoscopic needle aspiration in healthy humans (Ivarsson et al., 1998). This minimal invasive approach to collect mesothelial cells will however require further improvements to obtain clinically relevant numbers of cells for regenerative medicine applications. Additionally, the effluent peritoneal dialysis fluid from CAPD patients may also be considered as an alternative source of mesothelial cells, principally for heterologous cell therapies and tissue-engineering applications. However, in some instances these cells may have undergone partial to advanced myofibroblastic transdifferentiation, particularly when they are collected from long-term CAPD patients (Yanez-Mo et al., 2003; Zhang et al., 2013). The ability of mesothelial cells to transit between epithelial and mesenchymal phenotypes under specific *in vitro* culture conditions indicates that “myofibroblastic mesothelial cells” could be forced back to their original mesothelial phenotype and therefore potentially useful for the manufacture of bioartificial serosal mesothelial membranes.

### Parietal tunica vaginalis

The parietal tunica vaginalis (lamina parietalis), which is the parietal mesothelium of the testicular cavity, has been identified as another reliable source of mesothelial cells (Asano et al., 2005; Asano et al., 2006; Asano et al., 2007). It originates from an invagination of the peritoneum mesothelium that posteriorly descends into the scrotum. The parietal tunica vaginalis is a quite extensible membrane from which small pieces could be easily excised with minimal health concerns. The authors demonstrated the feasibility to harvest portions of 3.5 × 4.0 cm^2^ of tissue from each testis of beagles. Around 4.0 × 10^5^ mesothelial cells were obtained from each portion by using enzymatic digestion with Dispase I and could be successfully expanded *in vitro* to generate a confluent cobblestone-type monolayer of cells. Their subculture onto a fibrin gel could allow the generation of autologous mesothelial cells sheets. Interestingly, their apposition onto the surface of injured peritoneum areas (lacking mesothelium) could significantly improve their healing and reduce the score of peritoneal adhesions (Asano et al., 2006). On this basis, the authors suggested that small biopsies of parietal tunica vaginalis offer the advantage of representing an easy accessing and attractive therapeutic source of mesothelial cells, principally for patients with abdominal complications.

### Biomaterials useful for tissue engineering of simple squamous epithelia

Animal tissue-derived ECM proteins or purified natural polymeric molecules (proteins or polysaccharides) derived from plants or animals are highly sophisticated molecules that emerged from millions of years of natural evolution. They are usually endowed with desirable properties such as high degradability, excellent biocompatibility, and biomechanical properties such as elasticity, tensile strength, and transparency, which make them excellent candidate materials in biomedical engineering applications (Shin et al., 2003; Badylak, 2007; Ma, 2008).

Of further relevance, biopolymers-based scaffolds or acellular tissues usually provide a highly porous environment for cell invasion and excellent cell adhesion and growth properties (Velema and Kaplan, 2006). In many instances, natural polymers are also easy accessible and cheap to manufacture. Furthermore, and not less important, natural polymeric molecules are also usually rich in chemical side groups to which functionalizing molecules can be bound through chemical post treatments to generate hybrid biological scaffolds with improved cells adhesion, growth, and colonization outcomes.

To date, biological materials have been already used to manufacture biomimetics of the mesothelium, the vascular endothelium, or the corneal endothelium (detailed in further sections). We below review different types of biologic materials that have been previously proposed or used in tissue engineering of diverse types of simple squamous epithelia or related tissues (see also Table 2, for summary).

**Table 2:** **Biological laminar scaffolds potentially applicable for tissue engineering of human simple epithelia**.

Scaffolds	Basic component	Reference	Application
Native tissues [decellularized]			
Animal	Amniotic membrane Omental mesothelium Small intestinal mucosa Pericardium Cornea	Tsai et al. (2007) Hoganson et al. (2010) Andree et al. (2013) Dong et al. (2013) Feng et al. (2014)	VTE RM VTE, SE VTE Cornea, CE
Human	Amniotic membrane Cornea Lens Capsule	Wilshaw et al. (2006) and Kuriu et al. (2009) Feng et al. (2014) Lachaud et al. (2014b)	VTE, PA Cornea, CE CE.
Bioengineered sheets	Chitosan Alginate Silk fibroin+gelatin	Grolik et al. (2012) d’Ayala et al. (2008) Taddei et al. (2013)	CE SE SE
Hydrogels [compressed and/or cross-linked]	Gelatin Silk Fibroin+Gelatin Chitosan Chitosan+Collagen	Lai and Li (2010), Watanabe et al. (2011), and Lai et al. (2013) Grolik et al. (2012) Rafat et al. (2008) and Lai and Li (2010) Rafat et al. (2008)	CE, Cornea C. Epi. CE, Cornea CE, Cornea
Fibrous meshes [electrospun fibers]			
Simple	Silk Fibroin Collagen Laminin I	Liu et al. (2011), Madden et al. (2011), and Lv et al. (2014) Jiang et al. (2013) Neal et al. (2009)	VTE, SE, CE SE SE
Hybrid biologic	SF+Gelatin SF+Chitosan SF+Collagen IV SF+Fibronectin SF+Chondroitin−Laminin Keratin+Chitosan Chitosan+Laminins peptides Alginate+Laminins peptides	Feng et al. (2014) Guan et al. (2013a,b) Madden et al. (2011) Madden et al. (2011) Madden et al. (2011) Vazquez et al. (2015) Yamada et al. (2011) Yamada et al. (2011)	CE, Cornea Cornea CE CE CE Cornea SE SE
Hybrid bio-synthetic	SF+poly (ϵ-caprolactone)	Lv et al. (2014)	SE

*CE, corneal endothelium; C.Epi, corneal epithelium; ECM, extracellular matrix; SF, silk fibroin; PA, peritoneal adhesions; RM, regenerative medicine; SE, simple epithelia; VTE, vascular tissue engineering*.

### Decellularized animal tissues

The manufacture of humanized tissues with biological scaffolds derived from the decellularization of animal tissues has gained increased interest in recent years, principally because they provide many advantages over the use of artificial biomaterial scaffolds (Fu et al., 2014). Indeed, animal tissues of porcine, ovine, or bovine origin are easily available and cheap, and their decellularized forms are low immunogenic, bioactive, and biocompatible, and retain mostly original mechanical properties of native tissues. Not less important, animal tissues and most particularly those of porcine origin usually display similarities in size and histoarchitecture with their human counterparts. In light of this evidence are several studies that reported the usefulness of decellularized porcine tissues such as heart valves, arteries, dermis, tendons, or cornea in human clinical applications or in animal models research (Hoshiba et al., 2010; Klopsch and Steinhoff, 2012; Yoeruek et al., 2012).

The manufacture of humanized serosal membranes biomimetics is still in its early stages of development and as such only a limited number of works have already reported the use of animal-derived decellularized tissues for their manufacture. It may however be expected that they will be focus of broader interest in next future, particularly regarding to the use of the decellularized porcine epicardium, omental mesothelium, and mesentery. In support of this concept, a previous study indicated that a decellularized porcine mesothelium represents an optimal biological laminar scaffold, which preserves in most extent its original mechanical and biochemical properties (Hoganson et al., 2010). The decellularized porcine pericardium has also been proposed as potential biological scaffold for tissue engineering of heart valves (Dong et al., 2013). Additionally, the use of a decellularized porcine small intestinal submucosa has been focus of intense research for a variety of experimental tissue-engineering applications including skin, esophageal, cardiovascular, gastrointestinal, and musculotendinous regeneration [for review, see Andree et al. (2013)]. Its application in combination with human mesothelial cells should facilitate the manufacture of biomimetics of the vascular endothelium, the corneal endothelium or inclusively of the synovial intimal lining.

It is therefore reasonable to hypothesize that the use of porcine decellularized tissues such as the mesenteric membrane could be successfully combined with human mesothelial cells and potentially useful for the regeneration of simple squamous epithelia. This consideration will remain to be experimentally addressed.

### Decellularized amniotic membrane

The human amniotic membrane is a thin avascular membrane composed of a stromal layer and a basement membrane on which is anchored a layer of amniotic cells that are bath in amniotic fluid. The use of the amniotic membrane as potential scaffold in tissue engineering has attracted growing interest since many decades ago, principally because of its wide availability, large surface, and remarkable biological and mechanical properties of its matrix. At term, the human amniotic membrane reaches a total surface area ranging between 1300 and 1500 cm^2^ (Niknejad et al., 2008). An adequate decellularization process of the amniotic membrane could strongly minimizes the loss of its original bioactivity (growth factors) and mechanical properties of its stromal matrix and particularly those of its basement membrane, which is critical for an optimal cells adhesion and growth (Hopkinson et al., 2008). However, and soon before parturition, the ECM proteins of amniotic and chorionic membranes are rapidly broken down by matrix metalloproteinases (MMPs) to facilitate their mechanical rupture. The loss of their original mechanical properties may therefore potentially compromise their use for tissue-engineering applications where tensile strength is a critical factor. Amniotic membranes obtained from cesarean will overcome these limitations.

Decellularized amnion has been already used to build grafts that were experimentally applied for wound dressing, skin regeneration (Wilshaw et al., 2006), peritoneal adhesions (Kuriu et al., 2009; Yetkin et al., 2009), peritoneum reconstruction, vascular endothelium replacement (Tsai et al., 2007), or even more for corneal epithelium and endothelium replacement (Xu et al., 2012; Fan et al., 2013; Feng et al., 2014). Technical advances in decellularization processes have permit the generation of a commercially available decellularized and dehydrated human amniotic membrane (DDHAM) allograft which demonstrated positive clinical outcomes for healing of chronic wounds in humans (Smiell et al., 2015) and potential use in ocular surface surgery (Lim et al., 2010).

### Natural polymers and extracellular matrix proteins

Polymeric biomolecules produced either by plants or animals have attracted increasing interest for many tissue-engineering applications. Technological progresses made in the field of biomaterials have permitted that many of these natural polymeric molecules were better isolated, purified, chemically modified, and processed in the form of biological scaffolds (Shin et al., 2003; Ma, 2008). A host of natural biopolymers have been already tested to create thin laminar scaffolds applicable to tissue engineering of serosal membranes biomimetics. Cellulose is the best example of plant biopolymer potentially useful in biomedical research. Alginate, a large polysaccharide principally extracted from brown algae is also another good example of useful plant-derived biopolymer for biomedical applications. Regarding animal-derived biopolymers, the suitability of native polymeric ECM such as collagen, chitosan, or inclusively hyaluronic acid used alone or in combination in order to build biological laminar scaffolds is under intense focus and is detailed below.

#### Collagen

Collagen proteins are the predominant structural matrix components in connective animal tissues, representing an average of 25–35% of the total body weight. Collagen proteins are very diverse, and each of them endowed with unique properties [for review, see Kadler et al. (2007)]. Their excellent biocompatibility, bioactivity, degradability, and processing capacities into diverse solid formats (sheets, tubes, gels, or porous sponges) make of collagens excellent biomaterials to engineer tailored biological scaffolds and produce them on a large scale. Collagen type I is the predominant type of collagen used in tissue engineering, allowing the formation of rope-like structures that confer strength to bioengineered scaffolds. Collagen-based scaffolds used in tissue engineering of simple squamous epithelium biomimetics should be thin membranes with high collagen density and tensile strength to minimize their contraction by cell shrinkage. Ideally, this limitation can be strongly overcome by reinforcing collagen fibers cohesion by physical or chemical post-crosslinking (Fathima et al., 2010; Jiang et al., 2013) or through combination with other biological materials conferring additional strength such as silk fibroin (SF) (Madden et al., 2011). Collagen-based scaffolds subjected to post-crosslinking have been already used to bioengineer corneal endothelium biomimetics (Mimura et al., 2012).

#### Fibronectin

Fibronectin is a multifunctional glycoprotein actively involved in a variety of cells–cells and cells–matrix interactions through distinct classes of binding domains specific to integrins, growth factors, fibrin, heparin, collagen, glycosaminoglycans, and proteoglycans (Zhu and Clark, 2014). Fibronectin is a significant ECM component of the basal lamina in native simple epithelial tissues and in the endothelium (Laurie et al., 1982; Yen et al., 1997; Witz et al., 2001). Its role in tissue engineering has gained increasing force as functionalizing molecule increasing the bioactivity of biological scaffolds, principally since the discovery of its RGD binding domain, a tripeptide composed of L-arginine, glycine, and L-aspartic acid, also present in other ECM proteins such as collagen, that could be synthetically used as substitution of fibronectin for coating of material scaffolds and increase their cellular adhesion capacity (Pierschbacher and Ruoslahti, 1984).

#### Laminins

Laminins are key protein components of the basal lamina layer of basement membranes of distinct types simple epithelia (Laurie et al., 1982). As such, different tissue-engineering applications already integrated a laminin coating treatment of biological scaffolds as a critical step to enhance cells adhesion. Laminin coating of collagen compressed gels was already performed to create a bioartificial cornea (Mi and Connon, 2013). In a similar way, coating of prosthetic vascular small conduit grafts with laminin alone or in combination with collagen, considerably increased endothelial cells adhesion on the luminal graft surface (Chlupac et al., 2014). Of particular interest is the generation of a fibrillar mesh with electrospun laminin that under adequate conditions could generate a thin basement membrane-like carrier for cells adhesion and growth (Neal et al., 2009). The identification of laminin’s peptide sequences with cell-binding activities is of particular interest to develop synthetic basement membrane by covalently binding synthetic laminin peptides with polysaccharides such as chitosan or alginate (Yamada et al., 2011).

#### Gelatin

Gelatin is obtained through partial hydrolysis of collagen, leading to a mixture of peptides and amino acids. As gelatinous substance, gelatin is a highly malleable substance that could be cast in a large variety of 3D scaffold molds. Given to its excellent biocompatibility and malleability, gelatin is also commonly used in combination with solid biomaterials providing strength, while gelatin is used as complement to increase cell adhesion and growth, by providing a soft substrate closer to native ECM (Santoro et al., 2014). Gelatin is particularly useful to generate thin laminar scaffolds somehow mimicking native basement membranes and also to provide a carrier for cells sheet establishment and transplantation. In this way, gelatin hydrogels have been demonstrated useful for tissue engineering of a basement membrane biomimetic for the vascular endothelium (Bruggeman et al., 2012) and also for the corneal endothelium, through establishment of porous gelatin disk generated through cross-linking with carbodiimide (Lai and Li, 2010; Lai et al., 2013).

#### Alginate

Alginate or alginic acid is an anionic polysaccharide with high hydration capacity and which is widely distributed into brown algae (d’Ayala et al., 2008). Hydrated alginate can form a viscous gel highly compatible for cells and with high biodegradability. By controlling its processing, purified alginate powder can form a solid gel that is mainly useful for cells encapsulation procedures (d’Ayala et al., 2008). Alginate gels have however the downsides of displaying reduced mechanical strength and limited cell adhesion capacities. Like gelatin or inclusively collagens, alginate is generally mixed with other types of biomaterials with higher strength and cell adhesion properties such as chitosan or SF (Lai et al., 2007; Watanabe et al., 2011).

#### Chitosan

Chitosan is a long-chain polysaccharide derived by deacetylation of chitin, a polymer of N-acetylglucosamine, the structural component of the exoskeleton of arthropods and insects. Commercial chitosan is generally obtained from crustacean shells, mainly from shrimps and crabs. The high-abundance of chitin in nature, makes of its derivative, chitosan a very easy accessible and cheap biomaterial with several desired biological properties for tissue engineering of biological scaffolds, such as a high biocompability, easily processable by only pH modification, highly malleable (molding, casting) to generate scaffold with a desired form, porosity, and stiffness. Furthermore, and not less important, the polymer chitosan is particularly rich in chemical side groups, that allow covalent binding with other biomaterials to produce biodegradable biocomposites with increased strength and cell adhesion potential (d’Ayala et al., 2008). Mixtures of chitosan with other biopolymers such as SF or collagen represent useful biocomposites to create laminar biological scaffolds (Grolik et al., 2012; Guan et al., 2013a; Guan et al., 2013b). Additionally, chitosan has also been combined with synthetic polymers such as polyethylene glycol (PEG) for corneal tissue engineering (Rafat et al., 2008).

#### Silk Fibroin

Silk fibroin obtained from the silkworm (bombyx mori) is considered as a new bioengineering treasure by a majority of the biomedical community. Its outstanding biophysical and biochemical properties such as robustness, flexibility, biocompatibility, biodegradability, and processing properties have prompted a general interest for its use in tissue-engineering applications [for review, see Chen et al. (2011)]. The process of electrospinning allows the production of nano/micro SF fibers and their arrangement in a large varieties of scaffolds including aligned biofunctional nanofibers (Wittmer et al., 2011) and fibrous meshworks mimicking closely native ECM of a given type of tissue [for review, see Zhang et al. (2009)]. Interestingly, electrospun SF nanofibers can provide a high surface area improving cells surface adhesion, while maintaining high porosity for oxygen and molecules permeation, two of the properties provided by the basal lamina in serous membranes. Electrospun SF fibers can be alternatively post-treated with either synthetic or biologic compounds [hyaluronic acid, gelatin, chitosan, polycaprolactone (PC), polypyrrole, etc.] to generate hybrid scaffolds with increased properties or inclusively newly acquired functions (Huang et al., 2013; Yan et al., 2013).

Bioartificial functionalized SF fibrous scaffolds have been yet applied for tissue engineering of vascular endothelium and corneal endothelium biomimetics (Liu et al., 2011; Madden et al., 2011). In these works, the authors described how vascular and corneal endothelial cells (CECs) could readily adhere onto these constructs, proliferate, and form confluent monolayers of cells retaining original phenotypic characteristics. Despite such interesting results, SF-based fibrous meshes have still not been applied to the manufacture a mesothelial tissue biomimetic. Similar positive outcomes with the use of mesothelial cells are likely to be expected, given their close phenotypic similarities with vascular endothelial cells (Chung-Welch et al., 1989, 1997a,b) and CECs (Jirsova et al., 2010; Lachaud et al., 2014b).

## Mesothelialization Processes of Biological Scaffolds

The fabrication of a scaffold and its subsequent cellularization are critical steps conditioning the success of the resulting bioengineered tissue. Usually, different type of artificial biological scaffolds may be eventually post-treated with combined proteins generating an artificial basement membrane with are critical for the establishment of artificial simple epithelia. Their subsequent cellularization process is also complex and should basically take into account the phenotypic properties of the cells to be seeded and the cellular architecture of native tissues to be replaced.

The manufacture of simple squamous epithelia biomimetics with the use of mesothelial cells can be roughly achieved by using two distinct cellularization approaches, which can be either through classical static cell-seeding and culture of the scaffold or alternatively through culture of harvestable mesothelial cells sheets that are further layered on top of the scaffold (Asano et al., 2006).

### Precoating of biological scaffolds with an artificial basement membrane

The basement membrane is a thin multilayered matrix that acts as an anchoring substrate for the epithelium, mesothelium, and endothelium and represents a separating barrier from the underlying connective tissue. In some instances, some hybrid biological scaffolds (i.e., hybrid electrospun SF/polycaprolactone meshes), or scaffolds subjected to posterior treatments (cross-linking) to improve their mechanical properties, can however lose biological properties such as poor cells adhesion index and may require surface treatments with basement membrane proteins. In that sense, different tissue-engineering applications aimed at reproducing and fixing a synthetic basement membrane on top of biologic or synthetic scaffolds to improve adhesive and functional properties of their surface. As example, a study demonstrated that electrospun SF/polycaprolactone meshes posteriorly coated with major basement membrane proteins such as collagen IV, laminins, entactins, and proteoglycans could improve *in vivo* esophageal epithelium regeneration (Lv et al., 2014).

### Static cell-seeding

The cellular homogeneity and density achieved in a bioengineered mesothelium or in other types of simple squamous epithelial-like tissues are two critical parameters. The static cell-seeding technique, which basically consists in the manual pipetting of a concentrated cells suspension onto the scaffold, has been shown to be a convenient approach for the establishment of mesothelial cells layers (Takazawa et al., 2005; Asano et al., 2006; Kawanishi et al., 2013). Additionally, our laboratory recently reported that a suspension of mesothelial cells adequately dropped on top of human lens capsules, followed by their subsequent culture for a short period in a media enhancing their proliferation and blocking their epithelial-to-mesenchymal transition is reliable procedure to generate an efficient (homogeneous and cellularity) mesothelialization of their surface (Lachaud et al., 2014b).

### Cell sheet-based tissue engineering

The generation of non-invasive methods to detach intact cells monolayer with its deposited ECM has been made possible thanks to the development of thermoresponsive cell culture dishes, which are classical polystyrene Petri culture dishes grafted with the thermoresponsive polymer poly-*N*-isopropylacrylamide (PNIPAAm), this “smart polymer” is capable of hydrophobic to hydrophilic reversible transition in response to temperature changes. By only decreasing the culture temperature below its lower critical solution temperature (LCST) that is around 32°C in pure water, PNIPAAm become hydrophobic, and consequently force the release of cells monolayer from the dishes (Takezawa et al., 1990; Okano et al., 1995). The cell sheet technology (CST) is particularly useful to obtain intact cells monolayer along with their underlying organized extracellular matrix (ECM) without the need to use a supportive scaffold. Not less important, this technique provides monolayered cells with intact plasma membrane proteins (e.g., cell-surface receptors and ion channels) that otherwise should be severely damaged by the proteolytic activity of trypsin required for classical subculture of adherent cells (Huang et al., 2010a). Of further importance, cells in the detaching monolayer can self-retract, a process which leads to a certain compaction of cells sheets. This issue is particularly important for certain therapeutic applications such as corneal endothelium regeneration where the density of the replacement cells monolayer is a critical factor related to tissue functionality.

The generation of mesothelial cells sheets with thermoresponsive dishes has already been reported (Asano et al., 2006; Kawanishi et al., 2013; Inagaki et al., 2015). Additionally, this technology was also employed to manufacture corneal epithelium and corneal endothelium biomimetics (Hsiue et al., 2006; Lai et al., 2007; Kobayashi et al., 2013). The possibility to manufacture sandwiched layers of cultured cells may inclusively facilitate tissue engineering of complex multilayered artificial tissues such as artificial blood vessels or a bioartificial cornea, through applications of corneal epithelium and endothelium biomimetics on opposite sides of a bioartificial corneal stroma.

## Therapeutic Applications of Mesothelial Serosal Membranes Biomimetics

The use of mesothelial cells in regenerative medicine has gained increased interest over the course of the last decades. The clinical accessibility and plasticity of mesothelial cells, among other properties of these cells, have stimulated the interest of a significant number of researchers in evaluating their usefulness for diverse regenerative applications, which are reviewed below.

### Prevention of peritoneal adhesions

The secretion of lubricants by mesothelial cells provides a slippery, non-adhesive, and protective surface enabling visceral organs to freely move inside celomic cavities (Mutsaers and Wilkosz, 2007). A loss of mesothelium lining provoked either by surgery, ischemia, infection, and foreign bodies or by trauma can lead to adhesions between opposite injured surfaces and the formation of a connective band of fibrous tissue (diZerega and Campeau, 2001).

Post-operative peritoneal adhesions are very frequent in patients undergoing open abdominal surgery and can frequently provoke critical intestinal obstructions. In women, endometriosis is a major cause of pelvic adhesion that could lead to chronic pelvic pain and infertility. Peritoneal adhesions are generally very painful since they strongly limit organs from moving freely. The physical separation of damaged serosal with biodegradable adhesion barriers such as Seprafilm (hyaluronic acid + carboxymethylcellulose) represents the main clinical strategy to prevent or reduce post-operative peritoneal adhesions [for review, see Caglayan et al. (2014)].

The development of new adhesion barriers remains however under current research due to the partial effectiveness of the commercially available adhesion barriers. A host of experimental approaches involving pharmacologic treatments and anti-adhesive polymers or molecules promoting endogenous mesothelialization are also under intense research (Arung et al., 2011; Brochhausen et al., 2012). There is a clear consensus among researchers about the idea that an instantaneous regeneration of the injured mesothelial cells layer represents the ideal anti-adhesive strategy to avoid serosal membranes adhesions. Working in this direction, several studies showed the effectiveness of tissue-engineered mesothelial cells sheets in preventing experimentally induced intraperitoneal adhesions in animals (Takazawa et al., 2005; Asano et al., 2006; Kawanishi et al., 2013; Inagaki et al., 2015).

### Vascular grafts

Coronary and peripheral vascular occlusive diseases are among leading health problems worldwide. They currently require vascular bypass procedures, being autogenous veins the ideal replacement option as they show optimal engraftment and do not require immunosuppressive therapies (Thomas et al., 1988). Unfortunately, patients do not always present suitable healthy vascular tissues and are therefore subjected to an interposition of a prosthetic vascular graft. These artificial conduits are however prone to thrombotic occlusion as the materials [mainly polyethylene terephthalate, or Dacron, and polytetrafluorethylene (PTFE)] by which they are made cannot properly avoid platelet adhesion and they usually develop neointimal hyperplasia. Their poor patency is particularly increased in synthetic conduits with a luminal diameter lower than 8–10 μm [for review, see Kapadia et al. (2008) and Palumbo et al. (2014)]. None of the experimental antithrombogenic strategies tested so far could generate artificial small-diameter vascular grafts displaying successful long-term patency (Seifu et al., 2013). Ideally, prosthetic vascular grafts should be lined with autologous vascular endothelial cells to provide an optimal antithrombogenic luminal surface. However, autologous healthy vascular endothelial cells are not always easily accessible or available. These limitations have prompted a general interest in identifying whether other cellular phenotypes could display similar antithrombogenic activities.

Almost quite similar to the vascular endothelium (Tsuzuki, 2009; Hagensen et al., 2012), the mesothelium is also a specialized simple squamous epithelium that retains intrinsic regenerative capacities (Foley-Comer et al., 2002). The initial misidentification of human omentum-derived mesothelial cells cultures with microvascular endothelial cells (Knedler et al., 1989; Takahashi et al., 1989) led to the evidence that mesothelial and endothelial cells are not only morphologically similar but also display phenotypic similarities as evidenced by their common expression of specific endothelial and simple epithelial cells markers (Chung-Welch et al., 1989; Potzsch et al., 1990; Takahashi et al., 1991; Chung-Welch et al., 1997a,b). It was concluded that only the use of a comprehensive panel of endothelial and mesothelial cells markers could readily allow the distinction of one cell phenotype from another. Not less important, it has also become evident that human mesothelial cells partially display some of the functional features specific to endothelial cells such as the ability to produce the fibrinolytic enzyme tissue-type plasminogen activator (t-PA), urokinase plasminogen activator (Chlupac et al.), plasminogen activator inhibitor type-1 and type-2 (PAI-1 and PAI-2), and the procoagulant protein tissue factor (TF) (Sitter et al., 1996; Chung-Welch et al., 1997a; Ivarsson et al., 1998). Furthermore, and similar to vascular endothelial cells, mesothelial cells similarly synthesize prostacyclin (prostaglandin I2), a molecule inhibiting platelet activation and acting also as vasodilator (Van de Velde et al., 1986). The close similarities between both cell phenotypes thus led some researchers to test whether the mesothelial cell is a good cellular surrogate for vascular endothelium tissue engineering (Louagie et al., 1986; Bull et al., 1988; Bearn et al., 1992; Theuer et al., 1996; Verhagen et al., 1998; Sparks et al., 2002).

Interestingly, the work of Louagie et al. indicated that patches of mesothelium grafted into the anterior wall of the common iliac vein in dogs did not suffer major damage after several weeks of implantation. Two canine transplantation studies using prosthetic (Dacron) arterial grafts seeded with mesothelial cells however generated markedly divergent outcomes, suggesting that the adhesion capacity of mesothelial cells on this type of synthetic material is reduced and thus strongly influences their long-term patency (Bull et al., 1988; Bearn et al., 1992; Bearn et al., 1993). Other study inclusively indicated the luminal mesothelialization of small-diameter ePTFE vascular prostheses with omental mesothelial cells decreased their patency and increased neointimal formation respective to control unseeded prostheses interposed in the same dogs (Verhagen et al., 1998).

Despite these diverging results regarding the potential of mesothelial cells in vascular tissue engineering, it should be considered that Dacron- or ePTFE-based vascular scaffolds display limited adhesive properties for cells attachment (Sarkar et al., 2007). Therefore, mesothelial cells should be rather preferentially combined with vascular scaffolds of biological origin. Porcine decellularized arteries or veins represent useful candidates as they display morphometric similarity to their human counterparts. Furthermore, efficiently decellularized animal arteries retain almost intact native ECM and basement membrane ensuring optimal adhesion of cells onto their luminal surface, which consequently minimize their delamination in response to the strong shear stress variation proper to the arterial blood flow (Zhu et al., 2008a; Quint et al., 2011).

### Corneal endothelium

The corneal endothelium is the innermost layer of the cornea bathed in the aqueous humor of the eye’s anterior chamber. It is made up of a monolayer of CECs anchored on top of a basal membrane, the Descemet membrane. The corneal endothelium layer is critically required for the functionality of the cornea, since CECs are the cells that actively regulate the hydration state of the cornea by pumping out the excess of water in the cornea stroma into the aqueous humor (Bourne, 2003). At the same time, the corneal endothelium is a semi-permeable barrier allowing the transit of solute and nutrients from the aqueous humor toward more superficial layers of the cornea to nourish corneal stromal fibroblasts and corneal epithelial cells. Adult CECs are post-mitotic cells that almost lack endogenous regenerative capacities. Diverse studies have shown that the healthy corneal endothelium suffers an inexorable age-related decline in cellularity, which is compensated through hypertrophy of preexisting cells (Senoo and Joyce, 2000; Bourne, 2003). The corneal endothelium could suffer a critical loss of cellularity as a result of either physical or chemical damages or genetic disorders. A loss of corneal endothelium cellularity below a critical threshold leads to an inevitable loss of functionality and a subsequent accumulation of water into the corneal stroma and ultimately to the loss of vision (Bourne, 2003). The only effective clinical treatment available so far is the transplantation of a donor whole cornea or corneal endothelium layer. The important shortage of suitable donor corneas has stimulated the development of host of experimental tissue-engineering strategies to create a bioartificial corneal endothelium or inclusively to achieve the manufacturing of a whole bioartificial cornea [for review, see Mimura et al. (2013)]. Although the majority of these experimental approaches were performed with human CECs, several other studies also reported that CECs-like cells could be obtained through directed differentiation of stem cells populations such as neural crest stem cells, mesenchymal stem cells, and embryonic stem cells [for review, see Yuan and Fan (2015)]. Our laboratory recently reported that mesothelial cells isolated from the mouse visceral adipose tissue sharing many phenotypical similarities with mouse CECs (Lachaud et al., 2014b). Furthermore, we found that mesothelial cells could readily adhere onto decellularized epithelial side surface of human lens capsules. Mesothelial could actively proliferate and generate a compact cell monolayer mimicking some of the main morphological features of the native corneal endothelium (Lachaud et al., 2014b).

Further studies remain to address whether a mesothelial cells monolayer could also be established on other types of biological scaffolds fulfilling the main biophysical characteristics of the Descemet membrane such are transparency, strength, elasticity, and permeability to small molecules (i.e., nutrients). Thin membranes created from electrospun bombyx mori silk fibroin (BMSF) are transparent and were shown to support the growth of human corneal epithelial (HCE) cells (Hogerheyde et al., 2014). Additionally, biological scaffolds that were already tested with human CECs may also be suitable to establish a mesothelial cells monolayer. Among them are found chitosan-based membranes, donor Descemet’s membrane, cross-linked collagen matrix, human corneal stromal disks, gelatin hydrogel disks, acellular porcine corneal matrix, plastic compressed collagen, and decellularized human amniotic membrane [for review, see Mimura et al. (2012), Mimura et al. (2013), and Zavala et al. (2013)].

## Potential Therapeutic Applications of Mesothelial Serosal Membranes Biomimetics

The evidence that mesothelial cells share structural and biochemical markers with other types of simple epithelial-like cells (see Table 1) supports the idea that these cells might be useful for the regeneration of other types of simple epithelial-like tissues. Some possible applications are suggested below.

### Synovial membrane

The synovial membrane is a thin membrane lining the inner surface of the fibrous joint capsule, tendon sheaths, and bursae. The healthy synovium surface layer of cells (intima) is made up of two types of synoviocytes: “phagocytic or absorptive” macrophage-like cells (Type A) and “secretory” fibroblast-like synoviocytes (Type B), these later accounting for around 80% of the total intimal cells (Smith, 2011). Synovial cavities are filled with synovial fluid, a viscous lubricating fluid which forms from an ultrafiltrate of plasma and the secretion of lubricant molecules (mainly proteoglycan-4 and hyaluronan) secreted by fibroblast-like synoviocytes and chondrocytes (Smith, 2011). Distinct to a true epithelium, intimal surface synoviocytes are not tightly adhered to each other by junctional complexes and do not rest on a clearly well developed and continuous basement membrane, even if some basement membrane proteins such as collagen IV and laminin are expressed beneath the basal membrane of fibroblast-like synoviocytes (Pollock et al., 1990; Smith, 2011).

Interestingly, a careful revision of the literature provides evidence of phenotypic similarities between fibroblast-like synoviocytes and mesothelial cells. Hence, both cellular phenotypes are mesodermal epithelial-like cells which display abundant microvilli on their apical membrane, a structural feature that is commonly found in cells secreting fluid (Smith, 2011). In addition, they both abundantly secrete hyaluronan (Hesseldahl and Larsen, 1969; Yung and Chan, 2007; Koyama et al., 2008). Furthermore, both cell phenotypes also express the pan ­mesenchymal marker vimentin and the intercellular adhesion molecule β-catenin, CD54 (ICAM-1), N-cadherin, and cadherin-11 (Shibata et al., 1996; Agarwal et al., 2008; Kato et al., 2013; Lee et al., 2013; Lachaud et al., 2014a; Lachaud et al., 2014b). Similar to mesothelial cells, fibroblast-like synoviocytes are also antigen-presenting cells and as such they express detectable levels of MHC-II and costimulatory molecule CD40 (Valle et al., 1995; Yang et al., 2004; Kato et al., 2013).

The evidence of phenotypic similarities between fibroblast-like synoviocytes and mesothelial cells stated above could lead to the suggestion that mesothelial cells may represent a putative cellular surrogate of fibroblast-like synoviocytes and suggest thus that mesothelial cells could be potentially useful for the regeneration of the synovial membrane lining.

### Mesothelium lining of the Reissner’s membrane

The vestibular duct (scala vestibuli) is a small cavity filled with perilymph inside the cochlea of the inner ear. The scala vestibuli is separated from the scala media by a very thin membrane termed vestibular membrane or Reissner’s membrane. The Reissner’s membrane facing the scala vestibuli is covered by a monolayer of mesothelial cells. The opposite side facing the cochlear duct a cavity filled by endolymph is covered by an epithelium. A thin basal lamina separates the mesothelium and epithelium layers (Qvortrup et al., 1994). The Reissner’s membrane acts principally as permeable barrier to separate the perilymph from the endolymph. It allows a selective diffusion of solutes and nutrients from the perilymph to the endolymph that tightly controls endolymph/perilymph homeostasis. The rupture of the Reissner’s membrane caused either by physical trauma or inclusively due to an increased endolymph pressure (endolymphatic hydrops in Ménières disease) leads to severe hearing loss. To date, no experimental cell-based therapies or tissue-engineering applications have been proposed to reconstruct or substitute the damaged Reissner’s membrane. The use of autologous peritoneal mesothelial cells in combination with adequate biological matrices may hypothetically allow the development of tissue-engineered surrogates of the Reissner’s membrane.

## Glossary

**Allograft:** The transplant of an organ or tissue from one individual to another of the same species with a different genotype.

**Autologous cells:** Cells isolated from and transferred to the same individual’s body.

**Autologous transplantation:** Transplantation of cells or tissues, which are derived, stored, and later given back to the same person.

**Bioartificial tissue:** Biological tissue manufactured *in vitro* and mimicking the properties and functions of the native tissue to be replaced.

**Biocompatible material:** A material that is biologically tolerated by the surrounding tissue or organ in which it is transplanted.

**Biological scaffold:** A structural support of biologic origin that serves for cell attachment and subsequent tissue development.

**Biomaterials:** Materials used to construct artificial organs, rehabilitation devices, or prostheses and replace natural body tissues.

**Bioprosthetic:** Referring to prosthesis of biological origin that can be either native tissues or prosthesis manufactured from biological materials.

**Coelomic cavities:** Fluid-filled body cavities, which are lined by an epithelium derived from the mesoderm.

**Peritoneal dialysis:** Process by which the peritoneal membrane is used as ultrafiltration interface to eliminate blood waste products through the use of a hypertonic solution flowing into and out of the peritoneal cavity through.

**Corneal endothelium:** The single layer of simple squamous epithelial-like cells lining the inner surface of the cornea.

**Decellularization:** The process by which cells are removed from tissues or organs to obtain their extracellular components. It is mainly achieved through perfusion or immersion and the use of enzymes and/or detergents.

**Epithelial-to-mesenchymal transition:** The process by which epithelial cells undergo a transformation into cells with mesenchymal characteristics.

**Extracellular matrix (ECM)**: Structural molecules produced by cells and excreted to the extracellular space within the tissues and that provide cohesive structure to hold tissues together.

**Great omentum:** A large fold of the peritoneum hanging down from the stomach and containing abundant vasculature and perivascular adipose tissue.

**Heterologous transplant:** Cells, grafts, or tissues derived from an individual of a different species in which they are transplanted, being therefore antigenically dissimilar.

**Immunomodulation:** The adjustment of the immune response to a desired level through immunopotentiation, immunosuppression, or induction of immunologic tolerance.

**Immunosuppression:** Reduction of the immune response, generally through the use of drugs, active molecules, or cells to prevent grafts rejection or control autoimmune diseases.

**Lineage tracing study:** Identification and follow-up of all progeny of a single cell using different experimental strategies available for lineage tracing such as live-cell imaging, fluorescent reporter constructs, inducible gene expression, and inducible recombinases.

**Matrix:** The intercellular substance of a tissue or the tissue from which a structure develops.

**Mesoderm:** One of the three primary germ layers developing in the early embryo.

**Mesothelial cells:** A type of simple squamous epithelial cells lining the walls of celomic body cavities and visceral organs located inside.

**Mesothelioma:** Tumor arising from malignant transformation of mesothelial cells.

**Myofibroblast:** A fibroblastic cell with some contractile properties and that is usually considered an intermediate cell between fibroblasts and smooth muscle cells.

**Parietal mesothelial cells:** The mesothelial cells lining the parietal surface of serous body cavities.

**Polymeric molecules:** Molecules of high molecular weight generated through polymerization of smaller molecules (monomers). They are produced either by living organisms or chemically.

**Regulatory T cells:** A subtype of T lymphocytes with immunosuppressive properties and capacities to abrogate autoimmune diseases.

**Serosal membranes:** Membranes lining serous cavities and composed of a single layer of simple squamous epithelial resting on a basement membrane underneath.

**Simple squamous epithelium:** A simple epithelium composed by a single layer of thin and flat polygonal epithelial cells with secretive properties.

**Transdifferentiation:** The transformation of a differentiated somatic cell into another type without the need of reverting into an intermediate pluripotent state.

**Vascular endothelium:** The monolayer of cells lining the lumen of blood vessels.

**Visceral mesothelial cells:** Mesothelial cells covering visceral organs inside body cavities.

**Visceral organs:** Organs located within internal body cavities, especially those located within the abdomen.

## Conflict of Interest Statement

Christian Claude Lachaud, Abdelkrim Hmadcha, and Bernat Soria are inventors of the Patent (use of mesothelial cells in tissue engineering and artificial tissues, PCT/EP2014/061746) filed by the Fundación Progreso y Salud, Vissum Corporation and NewBiotechnic SA. Berta Rodriguez-Campins is an employee of NBT SA.
